# Antioxidant, Anti-Inflammatory and Anticancer Peptides from Extreme Marine Environments

**DOI:** 10.3390/antiox15050615

**Published:** 2026-05-13

**Authors:** Muhammad Zakariya, Eleonora Montuori, Gwendoline Kopp, Alessandro Coppola, Daniela Giordano, Stefano Bruno, Chiara Lauritano

**Affiliations:** 1 Department of Ecosustainable Marine Biotechnology, Stazione Zoologica Anton Dohrn, Via A.F. Acton 55, 80133 Napoli, Italy; zakariyakhan291@gmail.com (M.Z.); eleonora.montuori@szn.it (E.M.); gwendoline.kopp@szn.it (G.K.); alessandrocoppola@cnr.it (A.C.); 2 Institute of Biosciences and BioResources (IBBR), National Research Council (CNR), Via Pietro Castellino 111, 80131 Napoli, Italy; daniela-giordano@cnr.it; 3 Department of Food and Drug, University of Parma, Area Parco delle Scienze 23/a, 43124 Parma, Italy; stefano.bruno@unipr.it

**Keywords:** peptides, anticancer, deep-sea, cold seas, tropics

## Abstract

Marine organisms have proven to be excellent sources of bioactive natural products with potential therapeutic applications. To date, seventeen marine-derived molecules are on the market for the treatment of human diseases, mainly cancer. While multiple bioactivities of marine compounds have been consecutively reported, peptides represent promising candidates for these applications. This review focuses on peptides from marine organisms living in extreme marine environments, such as the deep ocean, polar regions, and tropical ecosystems. These are particularly promising for further bioprospecting, since their distinctive conditions have driven the evolution of unique biomolecules, as well as unique stability profile that can improve efficacy, shelf life, and performance under a wide range of industrial conditions. Ziconotide (Prialt), a neurotoxic peptide derived from the venom of a marine snail (*Conus* sp.) found at depths greater than 1000 m, is already commercially available for the treatment of severe pain. Recent technologies and computational tools are speeding up the discovery of new peptides and enzymes (very few from extreme environments). Overall, the review reports about eight peptides with anticancer properties from deep environments, nine, two and seven from polar habitats with antioxidants, anti-inflammatory and anticancer properties, respectively, and approximately ninety peptides from tropical waters (five antioxidant, thirty-five anti-inflammatory and fifty-four anticancer peptides). However, future studies in extreme environments will need to develop and apply sampling technologies, cultivation systems, as well as methods to assess efficacy, side effects and mechanisms of action, in vitro and in vivo.

## 1. Introduction

The ocean covers about 70% of the Earth’s surface and represents a vast reservoir for the bioprospecting of biologically active compounds. Over the past decades, marine organisms, which account for nearly half of global biodiversity, with more than 5 million animal species have emerged as promising sources of therapeutic and nutraceutical compounds, attracting increasing interest from both academia and the biopharmaceutical industry as valuable producers of bioactive metabolites to address the current shortage of innovative and potent drugs [1,2]. However, the publications on the pharmaceutical applications of marine natural products are a small share of those reporting newly discovered molecules each year. In fact, according to the MarinLit database, dedicated to marine natural products research, there are currently 44,675 known compounds (https://marinlit.rsc.org/; accessed on 5 May 2026) [3].

In recent years, marine organisms have emerged as an extraordinarily rich source of bioactive secondary metabolites, including lipids, carbohydrates, complex polyketides, toxins, terpenoids, alkaloids, cyclic peptides, and steroids with significant pharmacological activity [4]. The diverse chemistry of these compounds is also due to specific ecological conditions such as high pressure, salinity fluctuations, extreme temperatures and complex symbiotic interactions that induce the synthesis of structurally and functionally unique substances. In fact, this remarkable chemical potential of marine organisms is largely linked to the distinctive conditions of marine ecosystems, as adaptation to environments, which are more hostile than the terrestrial ones, has driven the evolution of sophisticated and advanced chemical communication systems [5,6]. Among marine environments, extreme marine environments defined as oceanic habitats characterized by severe physicochemical conditions are still largely unexplored and unexploited compared to the terrestrial ones, due to their poor accessibility and the lack of dedicated infrastructure for research [7]. Only very recent advancements in sampling methodologies and improved access to these areas, coupled with the development of omics technologies, have opened new perspectives for their bioprospecting. However, greater research efforts are needed to explore marine species across diverse oceanic zones to identify novel bioactive compounds including toxins with potential applications in the pharmaceutical and nutraceutical fields [8].

Currently there are seventeen marine-derived compounds on the pharmaceutical market (https://www.marinepharmacology.org/approved; accessed on 5 May 2026) [9], the majority for the treatment of cancer. Among those, there is a peptide originally isolated from marine organisms living in extreme conditions and now in use to treat human diseases. Ziconotide (Prialt^®^), approved by the Food and Drug Administration (FDA) in 2004, is a synthetic calcium channel-binding conotoxin isolated from the sea snail *Conus magus* living at about 1000 m depth, used for the treatment of severe pain. Extreme marine environments include deep-sea ecosystems, characterized by the absence of sunlight, high hydrostatic pressure and low temperatures; deep hypersaline anoxic basins (DHABs), marked by extreme salinity and pressure; polar seas, such as the Arctic and Antarctic Oceans, defined by persistently low temperatures, seasonal ice cover and extreme photoperiods; and hot environments including tropical marine regions, which, despite their high biodiversity, are subjected to intense physicochemical stressors including thermal stress, seasonal variability and frequent extreme weather events [10].

Deep sea (below depth of 1000 m) is often recognized as an extreme environment associated with the absence of sunlight and the presence of high hydrostatic pressure and low temperatures [10]. In addition, cold seep ecosystems represent another important type of deep-sea extreme habitat, where methane- and sulfide-rich fluids are released from the seafloor, supporting chemosynthetic microbial communities independent of sunlight. Deep-sea extremophiles can proliferate under these challenging conditions and have developed a variety of strategies to cope with these inhospitable environments: for instance, the production of extremozymes (for cold and thermal adaptation), salt tolerance and/or pressure tolerance and secondary metabolites which may have versatile biomedical applications [11]. With existing knowledge and technologies, scientists can retrieve microbial cells from several meters below the seafloor [12].

The polar seas comprising the Arctic Ocean and the Southern Ocean beyond the Antarctic Convergence represent two of the coldest marine environments on Earth [13]. In the coldest years, sea ice in these regions accounts for 13% of the surface of the planet, profoundly shaping global climate and ocean dynamics. The Arctic Ocean, located north of 57° latitude, is marked by dramatic seasonal light fluctuations, persistently frigid temperatures, severe winter extremes, and dynamic exchanges with warm Atlantic and Pacific waters [14]. Conversely, the Antarctic Ocean, extending between 60° South latitude and the Antarctic continent, is geographically and thermally isolated by the Antarctic Circumpolar Current. Strongly shaped by seasonal snow and ice cover, extreme photoperiods, and persistently low temperatures, Antarctica sustains a unique endemic marine biota [15]. Hot marine environments include several habitats such as geothermal hot springs, hydrothermal vents, and tropical regions. While extreme hot-spring and hydrothermal-vent sites host mostly thermophilic Archaea [16], whose enzymes are primarily exploited for high-temperature biotechnological applications [17], tropical marine environments stand out for their richness in bioactive molecules of interest, including peptides with antioxidant, anticancer, and anti-inflammatory activities.

Tropical regions, found around the equator, display an exceptional biodiversity, including corals, sponges, tunicates, algae, and their microbial symbionts. They are considered extreme because they experience intense physico-chemical stressors: intense heat or thermal stress (which can cause, for instance, coral bleaching), wet and dry seasons, quite frequent typhoons and hurricanes [18]. These regions, usually found around islands, have a monthly average water temperature of 18 °C and experience two seasons. The organisms living in these environments are producers of metabolites with antimicrobial, antioxidant, anti-inflammatory and anticancer properties [19,20].

In this context, this is the first review to focus on peptides from extreme marine habitats with antioxidant, anti-inflammatory, and anticancer activities. The following paragraphs will first briefly describe the most common activities observed and will then provide an in-depth overview of peptides from extreme environments that have shown antioxidant, anti-inflammatory, and anticancer activities, describing their specific mode of action in vitro and in vivo (when available). Previous reviews have addressed different but related topics, such as marine extremozymes for biofuel production and bioremediation [21], hydrolases [22], enzymes from polar regions [23] or other extreme environments [24], microorganisms from extreme environments [25], peptides/proteins from marine organisms in general [26] and biotechnological applications of bioactive peptides from marine sources [27].

Our state-of-the-art review is concerned with peptides from extreme marine environments which remain largely unexplored and have been investigated mainly in deep ocean environments, polar environments and tropical marine ecosystems. Various studies were based solely on sequence homology or predicted functions, and only a limited number of enzymes have been functionally characterized and tested for these activities. In particular, examples included a photolyase derived from the Antarctic microalga *Chlamydomonas* sp. ICE-L involved in DNA repair in mice exposed to UVB [28], a glutaminase- and L-asparaginase from Antarctic fungi [29,30] with anticancer activity, and a novel β-agarase isolated by macroalgae-associated bacteria with anti-inflammatory activity [31].

### 1.1. Antioxidants

Oxidative stress induced by reactive oxygen species (ROS) contributes to chronic non-communicable diseases (NCD), including cancer, diabetes, neurodegenerative disorders, hypertension, cardiovascular disease, atherosclerosis, and autoimmune diseases [32]. Antioxidants are compounds that inhibit or delay oxidation by neutralizing free radicals and are used clinically in several contexts [33]. They have been investigated or applied as supportive agents in cardiovascular diseases, neurodegenerative disorders such as Alzheimer’s and Parkinson’s diseases, and in the management of diabetes and its complications. They are also employed to reduce damage associated with chemotherapy and radiotherapy, to modulate inflammatory conditions, and to support eye health. Additional applications include hepatoprotection and the promotion of skin repair and wound healing [33].

Many small-molecule antioxidants have shown promise in preclinical studies, including vitamins (e.g., vitamin C and vitamin E), polyphenols (e.g., resveratrol and flavonoids), carotenoids (e.g., β-carotene and lycopene), thiols (e.g., *N*-acetylcysteine), synthetic antioxidants (e.g., probucol and edaravone), endogenous compounds (e.g., coenzyme Q10) and synthetic phenolic antioxidants, such as tert-butylhydroquinone (TBHQ) [34,35,36,37,38]. Overall, despite extensive investigation, most antioxidants have not translated into clinical applications. A key challenge associated with their use is the clear definition of effective versus toxic doses. Antioxidants are also widely used to prevent oxidation, extend the shelf life of foods, and stabilize formulations (food, cosmetic, and pharmaceuticals). Also in this context, the definition of toxic doses is crucial (some examples are reported in Table 1).

As shown in the tables below, the peptides isolated from extreme environments may offer new opportunities for antioxidant solutions.

Lipid peroxidation is one of the key agents in the aging process and is a pathophysiological driver for many diseases. Antioxidants can suppress peroxidation by donating an electron or a hydrogen atom to free radicals synthesized from unsaturated lipids, terminating chain reactions by scavenging singlet oxygen, inactivating metal catalysts, and eradicating radical intermediates or initiators [54].

The antioxidant activities of bioactive peptides can be dependent on the molecular size of the peptide and the hydrophobic and/or aromatic nature of the amino acids in the peptide sequences. Peptides with small molecular size can easily cross the intestinal barrier and produce biological effects compared to proteins or larger polypeptides. Hydrophobic amino acids Trp, Pro, Tyr, Lys, Leu, Val, and His play an important role in radical scavenging of peptides [55]. Peptides with aromatic amino acids and peptides with Tyr, His, Trp, and Met sequence also have high antioxidant functions [56].

### 1.2. Anti-Inflammatory Compounds

Inflammation is a physiological response of the immune system aimed at the protection of the body against pathogens, tissue damage, or harmful stimuli. This response is complex and tightly regulated and represents a fundamental process for the maintenance of homeostasis [57]. However, when it becomes chronic or degenerative, inflammation can contribute to the onset of various pathologies, including autoimmune, cardiovascular, metabolic, and neurodegenerative diseases and cancer [58,59]. The activation of the inflammatory process involves a dense network of cellular mediators including cytokines, chemokines, and transcription factors such as NF-κB and AP-1, which regulate the expression of pro-inflammatory genes [60,61]. The treatment of pathological inflammation is currently based on the use of nonsteroidal anti-inflammatory drugs (NSAIDs) and corticosteroids, which act by inhibiting cyclooxygenase (COX) and modulating the activity of the glucocorticoid receptors, respectively [62,63]. Scientific research is increasingly moving toward the discovery of new natural compounds with anti-inflammatory activity with high specificity and low toxicity [64]. The molecular mechanisms underlying the anti-inflammatory activity are complex and multifactorial. Some molecules act on the phosphorylation of IκBα, preventing the nuclear translocation of NF-κB and the corresponding transcription of inflammatory genes [65]. Others target the MAPK cascade, regulating the activity of p38, ERK, and JNK kinases, which are essential in the cellular stress response [65].

Several metabolites from extremophilic organisms exert significant antioxidant activity, reducing the formation of ROS and limiting oxidative stress, a known enhancer of inflammatory processes [66,67]. A review by Chen et al. [68] documented the isolation of approximately 250 bioactive compounds with anti-inflammatory activity from marine organisms. These compounds modulate various pathways, including NF-κB and MAPK and inhibit the production of pro-inflammatory cytokines such as IL-1β, IL-6, and TNF-α [68].

### 1.3. Anticancer Compounds

According to the World Health Organization (WHO), cancer is one of the leading causes of death worldwide (https://www.who.int/news-room/fact-sheets/detail/cancer; accessed on 5 May 2026) [69]. Cancer includes multiple diseases that can affect any part of the body, and many can eventually spread into other tissues causing metastases that are often lethal. According to WHO, in 2020 there were about 1.80 million deaths for lung cancer, 904,000 deaths for colon and rectum cancer, 760,000 deaths for liver cancer, 660,000 deaths for stomach cancer, and 666,000 deaths for breast cancer. The most common cancer type varies depending on the country under consideration; however, according to the WHO, cervical cancer is the most common in 23 countries [69]. Looking for new drugs with specificity against the various cancer types remains one of the major priorities of the drug discovery research community. Marine organisms can produce a plethora of molecules ranging from terpenes to steroids, alkaloids, polyketides, etc. Many of them have been shown to exert antiproliferative and anticancer properties. Some have reached the market and are currently in use.

As reported on the marine pharmacology website (https://www.marinepharmacology.org/), there are seventeen marine-derived drugs on the market, of which 9 are for cancers (including multiple myeloma, lymphoma, leukemia, lung and urothelial cancer typologies). In addition, 29 compounds are currently undergoing clinical trials, of which 26 are being investigated against cancers [9].

Marine compounds can induce cancer cell death or inhibit cell proliferation through several mechanisms, ranging from the inhibition of tubulin polymerization, to the disruption of mitotic spindle formation and induction of cell cycle arrest [70] to actin disruption, histone deacetylase (HDAC) inhibition, serine protease inhibition, induction of mitochondrial apoptosis, modulation of the cellular oxidative stress, and the modulation of the expression of oncogenic signaling pathways, such as PI3K/Akt, focal adhesion kinase (FAK)/extracellular signal-regulated kinase (ERK) and MAPK/ERK [71,72]. Angiogenesis is also a critical process for tumor cell proliferation, development, and metastasis. Therefore, inhibiting angiogenesis is considered an important anticancer strategy to suppress cancer growth and metastasis. Compounds that limit sun damage by screening ultraviolet (UV) radiation may also be broadly considered to exhibit anticancer activity, as UV exposure induces oxidative DNA damage and cyclobutane pyrimidine dimers (CPDs), contributing to photoaging and skin carcinogenesis [73].

## 2. Peptides from the Deep Ocean Environments

Deep ocean environments represent an underexplored resource of natural compounds with potential applications for human health. A depth below 1000 m is considered an extreme environment [7]. The extreme conditions of temperature, pressure, and low light have driven both benthic and planktonic organisms to evolve the production of unique compounds, many of which show promising potential for drug discovery, particularly in anticancer applications. Table 2 reports the list of peptides found in deep ocean environments described in the literature to date.

Interesting examples of peptides isolated from organisms living at great depths are the cyclic dipeptides diketopiperazines. They constitute a class of compounds possessing a highly stable and versatile six-membered piperazine core, making them privileged scaffolds in medicinal chemistry [74]. Typically biosynthesized from amino acids, they are regarded as secondary metabolites or byproducts of terminal peptide cleavage and primarily function as cell signaling molecules. These compounds exhibit a wide range of biological activities, including antitumor effects [75,76]. Due to their chiral, rigid and highly functionalized structures, diketopiperazines can bind to numerous receptors with high affinity, resulting in diverse biological activities [70]. Consequently, they represent attractive scaffolds for the discovery of new lead compounds and the rational design of novel therapeutic agents.

Zhang et al. [77] isolated seven diketopiperazines from the actinomycete *Nocardiopsis alba* strain SCSIO 03039 collected from the sediments of the Indian ocean (1000 m depth) [77]. Among the seven peptides, methoxyneihumicin showed cytotoxic activity against breast cancer MCF-7 cells and glioblastoma SF-268 cells, with IC_50_ values of 4.6 and 12.7 µM, respectively. In addition, a compound named XR334 exhibited a slight cytotoxicity against the MCF-7 and SF-268 cells, with an IC_50_ value of 22.0 and 22.6 µM, respectively. Although the authors did not elucidate the mechanism of action underlying cytotoxicity [77], the mechanisms of action of diketopiperazines are known to involve several key cellular processes, including the inhibition of tubulin polymerization leading to cell cycle arrest at the G2/M phase [19], the induction of mitochondrial apoptosis with the loss of mitochondrial membrane potential, the reduction in ROS production and the interference with PI3K/Akt and MAPK/ERK pathways [78].

Zhou et al. [79] isolated a sequential tristhiazole-thiazoline-containing cyclic peptide, marthiapeptide A, from the deep-sea actinomycetes *Marinactinospora thermotolerans* strain SCSIO 00652 [79]. Marthiapeptide A showed strong cytotoxicity against a panel of human cancer cells such as human glioblastoma cell line SF-268, the breast adenocarcinoma cell line MCF-7, the lung carcinoma cell line NCI-H460, and the human hepatocarcinoma cancer cell line HepG2, with IC_50_ of 0.38, 0.43, 0.47, and 0.52 µM, respectively [79].

Tian et al. [80] isolated two cyclic peptides called Microsclerodermins N and O from the deep-sea marine sponge *Pachastrella* sp., collected at a depth of approximately 1000 m. These compounds featured a rare p-ethoxyphenyl moiety within their macrocyclic scaffold, contributing to their bioactivity. Microsclerodermins N and O showed cytotoxicity against three human cell lines: the triple-negative breast cancer MDA-MB-231 line, the non-small cell lung cancer NCI-H460 line, and the glioblastoma SF-268 line. IC_50_ values were 0.54 μM on MDA-MB-231 cells, 0.67 μM on NCI-H460 cells, and 0.72 μM on SF-268 cells, while Microsclerodermins O showed a slightly lower potency, with IC_50_ of 1.2 μM on MDA-MB-231 cells, 1.8 μM on NCI-H460 cells, and 2.3 μM on SF-268 cells [80].

Sungsanpin, a 15-amino-acid peptide, was isolated from *Streptomyces* species isolated from deep-sea sediment and showed an inhibitory activity in invasion assays on lung A549 cancer cells [81] at concentrations of 5 and 50 µM. From the Rotating-frame Overhauser Effect Spectroscopy (ROESY) NMR spectrum and three-dimensional structure, Sungsanpin was shown to adopt a lasso peptide structure, in which eight amino acids (GFGSKPID) form a macrolactam ring, while a seven-residue tail (SFGLSWL) threads through the ring. Um et al. [81] investigated the mechanism of action of sungsanpin in A549 cells. Sungsanpin appeared to up-regulate the expression of tissue inhibitors of metalloproteinases (TIMP-1 and TIMP-2), which inhibit extracellular matrix degradation mediated by matrix metalloproteinases (MMPs) [81]. These results underscored the therapeutic potential of deep-sea-derived cyclic peptides and support further investigation into their mechanisms of action and selectivity profiles [80].

Three new N-methylated cyclic tetrapeptides were isolated from the deep-sea fungus *Arachnomyces bostrychodes*, collected from a cold seep chemosynthetic ecosystem, highlighting the potential of extreme marine environments as sources of structurally unique peptides. These compounds, identified as Cyclo-(N-methyl-L-Leu-L-Ile-N-methyl-L-Leu-L-Val), Cyclo-(N-methyl-L-Leu-L-Val)_2_, and Cyclo-(N-methyl-L-Leu-L-Ile)_2_, belong to a rare class of cyclic tetrapeptides containing two N-methylated amino acid residues. Despite structural features typically associated with enhanced stability and membrane permeability, these peptides did not show significant cytotoxic, antimicrobial, proteasome inhibitory, or anti-osteoclastogenic activities under the tested conditions, suggesting that their biological roles in cold seep ecosystems and their potential bioactivities require further investigation [82].

In a study on the deep-sea cold-seep-derived fungus *Talaromyces amestolkiae*, two peptide-based compounds, talarodipeptides A and B, were identified among a broader set of secondary metabolites, establishing once again the biosynthetic potential of microorganisms from extreme marine environments. These compounds are small cyclic dipeptides (diketopiperazine derivatives), structurally derived from amino acid precursors (e.g., P and F residues), and representing a simple yet relevant class of marine peptides. Despite their peptide nature, talarodipeptides A–B did not exhibit significant antibacterial or cytotoxic activities under the tested conditions, in contrast to other non-peptidic metabolites reported in the same study. Their occurrence nevertheless reinforces the role of cold seep ecosystems as underexplored reservoirs of peptide scaffolds, which may display ecological functions or bioactivities not captured by standard in vitro assays and thus need further studies [83].

Finally, as reported in Section 5, various peptides have been identified from the deep-sea *Rhodococcus* sp. I2R by using genome mining associated with mass spectrometry [84]. In particular, of the identified molecules, the fraction 5 and rhodoheptin mixture showed moderate antiproliferative activity at 500 µg/mL on human A375 melanoma cells [84].

**Table 2:** Peptides isolated from marine organisms living in deep environments showing anticancer properties. Abbreviations: MCF-7 stands for human breast cancer cells; SF-268 stands for human glioblastoma cells; NCI-H460 stands for hypotriploid human cells; HepG2 stands for human hepatocellular carcinoma cells; MDA-MB-231 stands for human breast cancer cells; A549 stands for human adenocarcinoma cells.

Protein Name/Peptide Sequence	Anticancer Activity	Active Concentration	In Vitro/In Vivo	Mechanism of Action	Reference
Fraction 5 and rhodoheptin mixture	Human A375 melanoma cells.	500 µg/mL	In vitro	-	[84]
Methoxyneihumicin	Human breast cancer cells MCF-7; Human glioblastoma cells SF-268	IC_50_: 4.6 µM; IC_50_: 12.7 µM	In vitro	-	[77]
XR334	Human breast cancer cells MCF-7; Human glioblastoma cells SF-268	IC_50_: 22.0 µM; IC_50_: 22.6 µM	In vitro	-	[77]
Marthiapeptide A	Human glioblastoma cells SF-268; human breast cancer cells MCF-7; typotriploid human cells NCI-H460; Human hepatocellular carcinoma cells HepG2	IC_50_: 0.38 µM; IC_50_: 0.43 µM; IC_50_: 0.47 µM; IC_50_: 0.52 µM	In vitro	-	[79]
Microsclerodermins N	Human breast cancer cells MDA-MB-231; Hypotriploid human cells NCI-H460; Human glioblastoma cells SF-268	IC_50_: 0.54 µM; IC_50_: 0.67 µM; IC_50_: 0.72 µM	In vitro	-	[80]
Microsclerodermins O	Human breast cancer cells MDA-MB-231; Hypotriploid human cells NCI-H460; Human glioblastoma cells SF-268	IC_50_: 1.2 µM; IC_50_: 1.8 µM; IC_50_: 2.3 µM	In vitro	-	[80]
Sungsanpin (1), 15-amino-acid peptide	Human adenocarcinoma cells A549	5 and 50 µM	In vitro	Inhibition of cell invasion	[81]

## 3. Peptides from the Polar Environments

In addition to deep ocean environments, several peptides have been reported from cold habitats for antioxidant, anti-inflammatory, and anticancer activity.

### 3.1. Antioxidants

Table 3 summarizes peptides from marine organisms living in polar environments that have been reported to possess antioxidant properties. A diketopiperazine, cyclo-(L-Pro-L-Tyr), and a linear peptide L-Tyr-L-Val-L-Pro-L-Leu were isolated from the cell-free culture supernatant of the Antarctic psychrophilic bacterium *P. haloplanktis* TAC125 with antioxidant activity (evaluated by a DPPH free radical scavenging assay) [85].

Other examples are represented by peptides encrypted within native proteins of organisms such as Antarctic krill, fish, and mollusks, and are released through enzymatic hydrolysis exhibiting structural characteristics that confer stability and activity to them.

Antarctic krill (*Euphausia superba*) protein hydrolysates yielded antioxidant peptides such as SLPY, QYPPMQY, and EYEA, with scavenging activities on DPPH (EC_50_ values of 1.18 ± 0.036, 1.547 ± 0.150, and 1.372 ± 0.274 mg/mL, respectively), HO^.^ (EC_50_ values of 0.826 ± 0.027, 1.022 ± 0.058, and 0.946 ± 0.011 mg/mL, respectively), and superoxide anion radical (EC_50_ values of 0.789 ± 0.079, 0.913 ± 0.007, and 0.793 ±0.056 mg/mL, respectively) analyses. These peptides also demonstrated reducing power, inhibition of lipid peroxidation, and protection against H_2_O_2_-induced DNA damage [86]. The same krill peptides also showed ACE-inhibitory activity (SLPY: IC_50_ 0.3715 mg/mL; QYPPMQY: IC_50_ 0.2903 mg/mL; EYEA: IC_50_ 0.3375 mg/mL), ameliorating hypertension via significantly inhibiting ACE activity and protecting HUVECs against oxidative damage [87]. The mechanism revealed that SLPY, QYPPMQY, and EYEA activated the Keap1/Nrf2 pathway in HUVECs by significantly up-regulating Nrf2 protein expression and its nuclear level. The Keap1/Nrf2 pathway is a central endogenous antioxidant defense system and a key regulator of antioxidant molecules. It controls the expression of antioxidant factors and detoxification enzymes, enabling the timely and efficient removal of excess ROS [88]. SLPY, QYPPMQY, and EYEA were shown to up-regulate intracellular Nrf2 protein levels and promote the dissociation of Nrf2 from Keap1, facilitating its translocation into the nucleus. However, molecular docking analyses indicated that these peptides do not bind to the active sites of Keap1, suggesting that they activate the Keap1/Nrf2 pathway by promoting Nrf2 nuclear translocation through a non-competitive mechanism. This activation subsequently enhanced the activity of downstream proteases, promoted NO production, and reduced ROS, MDA, and LDH levels, decreasing oxidative stress in H_2_O_2_-damaged HUVECs and reinforcing their potential as nutraceuticals and therapeutic agents for oxidative stress-related disorders [88]. Other krill-derived peptides, particularly LKPGN and LQP [89] demonstrated significant radical scavenging capacity against hydroxyl, DPPH, and superoxide radicals. In cellular assays, LKPGN and LQP effectively protected Chang liver cells from H_2_O_2_-induced oxidative stress by enhancing cell viability and reducing apoptosis rate. They increased antioxidant enzyme activities (SOD, GPX), reducing reactive oxygen species (ROS), increasing mitochondrial membrane potential and lowering lipid peroxidation and DNA damage.

The Antarctic krill peptide SSDAFFPFR significantly suppressed the decrease in SOD activity and decreased ROS content induced by scopolamine in PC12 cells, as well as inhibited the expression of Bax, Caspase-3 and p53 and promoted the expression of BCL-XL, thereby protecting PC12 cells from the effects of oxidative stress [90]. The Antarctic krill peptide FPF derived from the antioxidant-rich Antarctic krill peptide SSDAFFPFR, reduced ROS and MDA levels, and increased SOD activity and unsaturated lipid levels in mice, decreased the expression level of Caspase-3 in mice and improved energy metabolism in HT22 cells [91].

Collagen peptides derived from the Antarctic jellyfish *Diplulmaris antarctica* represent another example of bioactive polar marine peptides. In a model of high-calorie diet-induced obesity, oral administration of low-molecular-weight collagen peptides (<10 kDa) reduced body weight gain and body mass index, improved markers of insulin resistance, and restored antioxidant status by increasing SOD activity and reducing levels of oxidatively modified proteins [92].

**Table 3:** Peptides isolated from marine organisms living in polar environments showing antioxidant properties. Abbreviations: ROS stands for reactive oxygen species, SOD for superoxide dismutase ACE inhibitors stand for Angiotensin-converting-enzyme inhibitors; HUVECs stand for Human umbilical vein endothelial cells; Keap1 stands for Kelch-like ECH-associated protein 1, and Nrf2 stands for nuclear factor erythroid 2-related factor 2; GPX stands for glutathione peroxidase; PC12 cells stand for a cell line derived from a pheochromocytoma of the rat adrenal medulla.

Peptide Sequence	Activity	Active Concentration	In Vitro/In Vivo	Mechanism of Action	Reference
Cyclo-(L-Pro-L-Tyr) and L-Tyr-L-Val-L-Pro-L-Leu	Antioxidant activity evaluated by a DPPH free radical scavenging assay	10 mmol–1 mmol	In vitro	-	[85]
SLPY, QYPPMQY, and EYEA	ACE-inhibitory with protection of HUVECs against oxidative damage	SLPY: IC_50_ 0.3715 mg/mL, QYPPMQY: IC_50_ 0.2903 mg/mL and EYEA: IC_50_ 0.3375 mg/mL	In vitro	Keap1/Nrf2 pathway in HUVECs by significantly up-regulating Nrf2 protein expression and its nuclear level	[87]
LKPGN and LQP	Protection of Chang liver cells from H_2_O_2_-induced oxidative stress	-	In vitro	Increase in antioxidant enzyme activities (SOD, GPX), reduction in ROS, increasing mitochondrial membrane potential and lowering lipid peroxidation and DNA damage.	[89]
Antarctic krill peptide SSDAFFPFR	Pheochromocytoma PC12 cells	-	In vitro	Suppression of the decrease in SOD activity and decreased ROS content induced by scopolamine, as well as inhibition of the expression of Bax, Caspase-3 and p53 and promotion of the expression of BCL-XL	[90]
FPF from an antioxidant-rich Antarctic krill peptide SSDAFFPFR	Mouse hippocampal neuronal HT22 cells; mice		In vitro; in vivo	Reduced ROS and MDA levels, and increased SOD activity and unsaturated lipid levels in mice, decreased the expression level of Caspase-3 in mice and improved energy metabolism in HT22 cells	[91]

### 3.2. Anti-Inflammatory Activity

Mass spectrometry-guided isolation revealed the serine dipeptide lipopeptide Lipid 430, produced by two *Algibacter* sp. strains of marine origin isolated from invertebrate-associated microbiota in the Barents Sea, specifically from the bryozoan *Alcyonidium gelatinosum* and the soft coral *Gersemia rubiformis* [93]. Beyond its antibacterial activity, Lipid 430 is structurally related to bioactive serine-dipeptide lipids known to modulate innate immune signaling. It acts as a ligand of Toll-like receptor 2 (TLR2), a key receptor in inflammatory responses, thereby modulating inflammatory signaling and exerting a context-dependent anti-inflammatory effect. Table 4 summarizes peptides showing anti-inflammatory activities.

High Fischer ratio oligopeptides from Antarctic krill *Euphausia superba* (HFOPs-AK) have demonstrated significant hepatoprotective effects in a mouse model of alcoholic liver injury [94]. HFOPs-AK improve liver and kidney histology, reduce serum markers of hepatocellular damage (ALT, AST), correct alcohol-induced dyslipidemia, and strongly suppress inflammation by decreasing TNF-α and IL-6 levels, up-regulating IκBα, and inhibiting NF-κB activation. These oligopeptides also enhance antioxidant defenses, reduce oxidative stress, suppress CYP2E1 activity, and regulate lipid metabolism through AMPK/Nrf2/IκBα signaling pathways [94].

**Table 4:** Peptides isolated from marine organisms living in polar environments showing inflammatory properties. Abbreviations: TLR2 for ligand of Toll-like receptor 2; ALT for Alanine Aminotransferase; AST for Aspartate Aminotransferase.

Peptide Sequence	Activity	Mechanism of Action	Reference
Serine dipeptide lipopeptide Lipid 430	Modulate innate immune signaling, serves as a ligand of Toll-like receptor 2 (TLR2)	-	[93]
HFOPs-AK (Oligopeptides)	Improve liver and kidney histology, educe serum markers of hepatocellular damage (ALT, AST), correct alcohol-induced dyslipidemia	Suppress inflammation by decreasing TNF-α and IL-6 levels, up-regulating IκBα, and inhibiting NF-κB activation, suppress CYP2E1 activity, and regulate lipid metabolism through AMPK/Nrf2/IκBα signaling pathways	[94]

### 3.3. Anticancer Activity

Table 5 presents the peptides isolated from marine organisms inhabiting polar environments that exhibit anticancer activity.

A rare bioactive diketopiperazine, cyclo-(L-Pro-L-Met) identified in the marine actinomycete *Nocardiopsis* sp. 03N67, isolated from the seaweed *Undaria pinnatifida* in the Arctic region, exhibited anti-angiogenic activity against human umbilical vein endothelial cells (HUVECs) [95]. Cyclo-(L-Pro-L-Met) inhibited TNFα-induced tube formation and invasion at 10 mM, a concentration at which no cytotoxicity was observed. The anti-angiogenic activity exhibited by cyclo-(L-Pro-L-Met) underscores its potential as a bioprobe for the rational design and development of novel anticancer therapeutics based on small-molecule scaffolds [95].

A cytotoxic polypeptide (MW: ~19 kDa), PBN11-8, purified from the fermentation broth of an Antarctic marine *Bacillus* sp. N11-8, displaying high similarity to peptidase M84 from *Bacillus pumilus*, possessed moderate cytotoxicity towards several cancer cell lines with IC_50_ values of 1.56, 1.80, 1.57, and 1.73 mg/mL against human hepatocellular carcinoma cell line BEL-7402, human renal clear cell adenocarcinoma cell line 786-0, human hepatocellular carcinoma cell line HepG2, and human pancreatic cancer cell line Panc-28, respectively [96]. Wound-healing migration and transwell assays demonstrated that PBN11-8 effectively inhibited the migration and invasion of BEL-7402 cells. Further mechanistic analysis revealed that PBN11-8 suppresses FAK-mediated adhesion, migration, and invasion by disrupting FAK/ERK signaling and down-regulating matrix metalloproteinases MMP-2 and MMP-9 in BEL-7402 cells. Collectively, these findings indicated that PBN11-8 represents a promising lead compound for the development of novel anticancer agents [96].

Three cyclic acylpeptides, named as mixirins A, B and C, from marine bacterium *Bacillus* sp., obtained from sea mud near the Arctic pole, exhibited antitumor activities, inhibiting the growth of human colon tumor cells (HCT-116) with IC_50_ of 0.68, 1.6, 1.3 mg/mL, respectively [97].

A homologous peptide of NK-lysin, an antimicrobial peptide originally identified in mammals, has been identified in the head kidney transcriptome of the Antarctic teleost *Trematomus bernacchii* [98]. Two synthetic peptides, NKL-WT (KLKSKLMVVCNKIGLLKSLCRKFVKSH) and NKL-MUT (KLKSKLMVVANKIGLLKSLARKFVKSH) showed anticancer activity. Notably, NKL-MUT possesses improved cytotoxicity and higher pro-apoptotic activity compared to NKL-WT towards the melanoma cell line (B16F10), with no significant side effects on fibroblasts (FB789), at all the analyzed concentrations (40 and 80 µM). Upon exposure to melanoma cells, NKL-MUT triggered DNA fragmentation and apoptosis, supported by cell shrinkage, plasma membrane ruffling, and the disintegration of cell fragments into apoptotic bodies. Additionally, secondary necrosis characterized by the gradual loss of plasma membrane integrity in apoptotic cells was also detected in tumor cells [98].

**Table 5:** Peptides with therapeutic potential isolated from marine organisms living in polar environments with anticancer activity.

Peptide	Activity	Active Concentration	In Vitro/In Vivo	Mechanism of Action	Reference
Diketopiperazine, cyclo-(L-Pro-L-Met)	Anti-angiogenic activity in human umbilical vein endothelial cells (HUVECs)	10 µM	In vitro	Inhibition of TNF-α-induced tube formation and invasion	[95]
Mixirins A, B and C	Inhibition of the growth of human colon tumor cells HCT-116	IC_50_ 0.68, 1.6, 1.3 mg/mL	In vitro	-	[97]
Polypeptide PBN11-8	Cytotoxicity towards human hepatocellular carcinoma cell line BEL-7402, human renal clear cell adenocarcinoma cell line 786-0, human hepatocellular carcinoma cell line HepG2, and human pancreatic cancer cell line Panc-28	IC_50_ 1.56, 1.80, 1.57, and 1.73 mg/mL	In vitro	Suppression of focal adhesion kinase (FAK)-mediated adhesion, migration, and invasion by disrupting FAK/extracellular signal-regulated kinase (ERK) signaling and down-regulating matrix metalloproteinases MMP-2 and MMP-9 in BEL-7402 cells	[96]
NKL-WT (KLKSKLMVVCNKIGLLKSLCRKFVKS) and NKL-MUT (KLKSKLMVVANKIGLLKSLARKFVKSH)	Cytotoxicity and pro-apoptotic activity to melanoma cell line (B16F10)	40 and 80 µM		DNA fragmentation and apoptosis and secondary necrosis	[98]

## 4. Peptides from the Tropical Environment

Finally, another extreme environment for which peptides have been reported is characterized by tropics which are characterized by high temperatures, increased humidity and altered precipitation [99]. Compared to other extreme environments, tropical environments emerged as the most peptide-rich. Tropics are environments well known to be rich in biodiversity. Why so many species? There is still discussion about that, with many ‘historical’ and ‘ecological’ hypotheses recently centering mainly around phylogenetic niche conservatism and ecological productivity [18]. However, there is still little consensus. This very rich biodiversity in terms of species also corresponds to multiple molecules produced by marine organisms for defense and communication purposes. In addition, considering that it is easier to access to marine resources in tropics, compared to deep or ice-covered environments, several studies have shed light on the tropical therapeutic chemical diversity [18].

### 4.1. Antioxidants

Two fish species, *Nemipterus japonicus* and *Exocoetus volitans*, collected from Royapuram Fishing Harbor in North of Chennai, India, were used to produce antioxidant peptides by muscle enzymatic hydrolysis with three distinct enzymes: pepsin, trypsin, and papain. The highest antioxidant activity was found in trypsin hydrolysates with a value of 64% for *N. japonicus* and 65.8% for *E. volitans*. This hydrolysate was then fractionated, and the two most active fractions (fraction II for *N. japonicus* and fraction III for *E. volitans*) were further fractionated. The antioxidant radical scavenging for all fractions was evaluated by DPPH, hydroxyl radical scavenging and superoxide radical scavenging, finding scavenging activity between 40 and 50% [100].

In a study by Sudhakar and Nazeer [98], the Indian squid *Loligo duvauceli*, collected from the Royapuram, was used to evaluate the antioxidant activity of peptides. The hydrolysis of *L. duvauceli* mantle was performed using three different enzymes: trypsin, α-chymotrypsin and pepsin. The antioxidant potential of the active hydrolysates was evaluated with DPPH, hydroxyl, superoxide radical scavenging assays, metal chelating activity and reducing power ability. Although all three hydrolysates had an antioxidant activity, the α-chymotrypsin hydrolysate showed the best activity. Results showed DPPH radical scavenging (24.18 ± 0.33) at 1.5 mL/mL, hydroxyl radical scavenging (30.52 ± 0.82) and superoxide radical scavenging (32.47 ± 0.68%) at 250 µg/mL. However, the derived α-chymotrypsin hydrolysate exhibited an activity of 34.54 ± 0.7 for metal chelating activity and an activity of 0.141 ± 0.008 for reducing power [101].

In a study by Leticia Olivera-Castillo and collaborators, sea cucumber (*Isostichopus badionotus*), collected off the coast of Telchac Puerto, Yucatan, Mexico, was used to produce collagen peptides from the body wall. Collagen was solubilized with pepsin and subsequently digested with papain. The supernatant was fractionated using a membrane with cut-offs of 1 and 3 kDa obtaining collagen fractions of 1–3 kDa and >3 kDa. The 1–3 kDa fraction was subsequently fractionated by flash chromatography, finding 3 peaks. All fractions were tested for their antioxidant activity by ABTS and ORAC assays. The most active fraction was found to be the 1–3 kDa fraction and 1–3 kDa Peak 2. The 1–3 kDa fraction measured approximately 150 nmol TE/mg in both the ORAC and ABTS assays, while the 1–3 kDa Peak 2 fraction measured approximately 125 nmol TE/mg in the ABTS assay and approximately 250 nmol TE/mg in the ORAC assay [102].

The swim bladder of *Totoaba macdonaldi* was also used for collagen extraction using the classical method. The extracted collagen was digested with alcalase (HCA) and papain (HCP). The ultrafiltered fractions (≤3 kDa) of the collagen hydrolysates were tested at 3.2 mg/mL for their antioxidant activity. The DPPH assay showed an antioxidant activity (% of scavenging activity) of approximately 30% for HCP compared to approximately 40% for HCA [103]. Table 6 enlists peptides and fractions from tropical marine environments with antioxidant activity.

**Table 6:** Peptides/Fractions with antioxidant activity from tropical environments. Abbreviations: DPPH for 2-diphenyl-1-picrylhydrazyl radical scavenging assay.

Protein/Peptide Sequence/Fractions	Activity	Percentage Activity/Active Concentration	In Vitro/In Vivo	Reference
Trypsin hydrolysate; Fraction II	Lipid peroxidation inhibition	~64%	In vitro	[100]
Trypsin hydrolysate; Fraction III	Lipid peroxidation inhibition	~65.8%	In vitro	[100]
α-chymotrypsin hydrolysate	DPPH radical scavenging	24.18 ± 0.33%; 1.5 mg/mL	In vitro	[101]
α-chymotrypsin hydrolysate	Superoxide radical scavenging	32.47 ± 0.68%; 250 µg/mL	In vitro	[101]
Collagen hydrolysate (Alcalase, ≤3 kDa; HCA)	DPPH radical scavenging	~40%; 3.2 mg/mL	In vitro	[103]
Collagen hydrolysate (Papain, ≤3 kDa; HCP)	DPPH radical scavenging	~30%; 3.2 mg/mL	In vitro	[92]

### 4.2. Anti-Inflammatory Peptides

Table 7 compiles peptides with anti-inflammatory properties derived from marine organisms adapted to tropical environments. A 90-amino-acid residue lectin polypeptide, *Hypnea cervicornis* agglutinin (HCA), which was isolated from a red alga species, *Hypnea cervicornis*, found along the North-East Brazilian Coast, was discovered to reduce carrageenan-induced paw edema and inhibit neutrophil (neutrophils are the first responders to inflammation stimuli and which contribute to its resolution) migration in rats up to 90% at 10 mg/kg [104].

In Taori et al. [105], three analogues of dolastatin 13, a depsipeptide, and somamide B, a cyclodepsipeptide, were isolated from a marine cyanobacterium, *Lyngbya* sp., from South Florida. The four compounds exhibited strong inhibition of pancreatic elastase—a driver of inflammation [105,106] with IC_50_ values from 3 to 10 nM. Other analogues of dolastatin 13 were isolated from *Lyngbya* sp., such as Lyngbyastatin 4, a depsipeptide found to inhibit elastase with IC_50_ values of 0.03 μM [107]. Lyngbyastatins 5–10 inhibited porcine pancreatic elastase with IC_50_ values ranging from 3 to 210 nM [105,108]. A cyclodepsipeptide, kempopeptin A, also showed potential anti-inflammatory activity by inhibiting elastase with an IC_50_ of 0.32 μM [109].

Interleukin-5 is a pro-inflammatory cytokine that induces the activation of eosinophils which plays a key role in allergic inflammation and asthma [110]. Oh et al. [111] investigated the immunosuppressive properties of two new cyclic peptides, thalassospiramides A and B, that were isolated from a new member of the marine α-proteobacterium *Thalassospira*. Thalassospiramide B inhibited interleukin-5 production with an IC_50_ of 5 μM for thalassospiramide B, resulting in potential anti-inflammatory activity [111].

Three diketopiperazines were isolated from marine-derived bacteria from Jeju Island in South Korea (*Bacillus* sp. HC001 and *Piscicoccus* sp. 12L081), and found to be potent agents targeting TGFBIp to treat severe vascular inflammatory disease [112]. Two diketopiperazines were isolated from *Bacillus* sp. HC001: cyclo (L-Pro-D-Val) and cyclo (L-Pro-L-Tyr), and one from *Piscicoccus* sp. 12L081: cyclo (L-Pro-D-Leu). An in vitro study highlighted the inhibition of TGFBIp by the three diketopiperazines at an optimal concentration of 10 μM stimulated with LPS, similar results were found in in vivo study with mice treated with 10 μM.

Two cyclic peptides, including an aspochracin-type cyclic tripeptide sclerotiotide L (cyclo-(Thr-O-MeTyr-N-MeAla-Ile)) and a diketopiperazine dimer, were isolated from the tropical marine sponge-derived fungus *Aspergillus violaceofuscus* [113], and showed activity on the cytokine IL-10 expression of the LPS-induced THP-1 cells with 84.3 and 78.1% inhibition rate at 10 μM.

New peptides named acrepeptins A (N-Ac-Gln-Ile-N-Me-Ile-Ile-NMe-Val-N-Me-Ile-N-MeIle-N-Me-Leu) and C (N-Ac-Gln-Ile-N-Me-Ile-Ile-N-Me-Ile-N-Me-Ile-N-MeIle-N-Me-Leu) showed inhibitory activities on nitric oxide production in LPS-induced microglial BV-2 cells with IC_50_ values of 12.0 and 10.6 μM, respectively. These new peptides were isolated from the subtropical region of Taiwan from the red alga *Mastophora rosea*-derived fungal strain *Acremonium* sp. NTU492 [114].

Largazole, a cyclodepsipeptide, known to be a potent histone deacetylase inhibitor (HDACis), which was isolated from a tropical cyanobacterium of the genus *Symploca*, was characterized by Bhansali et al. [115] and studied by Ahmed et al. [116] for its modulation of several inflammation pathways. Indeed, treatment of rheumatoid arthritis (RA) synovial fibroblasts with the compound at 5 μM inhibited TNF-α-induced matrix metalloproteinase-2 (MMP-2) activity by up to 35%, a key factor in joint destruction in RA. This effect was accompanied by inhibition of TNF-α-induced nuclear translocation of NF-κB p65, which is associated with MMP-2 regulation.

From the Floridian cyanobacterium *Okeania lorea*, two linear depsipeptides, grassystatins A and B were isolated and found to have modulated inflammatory activities [117] against two proteases, cathepsin D and E, which are involved in anti-inflammatory activity by promoting neutrophil apoptosis and clearing damaged tissue for cathepsin D and pro-inflammatory activity by enhancing the production of pro-inflammatory cytokines for cathepsin E. Grassystatin A inhibited cathepsins D and E with IC_50_ values of 26.5 nM and 886 pM, respectively. Grassystatin B also inhibited cathepsins D and E with IC_50_ values of 7.27 nM and 354 pM, respectively.

An analogue of dolastatin 13, molassamide, which contains 6 common amino acids (Val-N-Me-Tyr-Phe-Ahp-Abu-Thr), was isolated from a cyanobacterial assemblage of *Dichothrix utahensis* collected from Florida and showed protease-inhibition activity against elastase with IC_50_ value of 0.032 μM [118].

Two other depsipeptide analogues of dolastatin 13 are bouillomides A (Val-N-Me-Tyr-Phe-Ahp-Abu-Thr-Val-Ala-Ba) and B. These two have the same amino acid sequence but the N-Me-tyrosine unit from bouillomide A is replaced by a brominated N-Me-Tyr unit. They were isolated from *Lyngbya bouillonii* [119], from Guam, an island with tropical weather. Both depsipeptides were found to inhibit elastase with an IC_50_ value of 1.9 μM [119].

Didemnin A and B also belonging to the class of cyclic depsipeptides, were isolated from the Caribbean tunicate *Trididemnum solidum* [120], which is of great interest for its anticancer properties. These molecules were found to show anti-inflammatory activity by inhibiting the inducible nitric oxide synthase (iNOS) and the nuclear factor-kappa B (NF-κB) activity, with an IC_50_, respectively, of 0.2 and 0.002 µM and 0.2 and 0.03 µM.

In the study of Festa et al. [121], two cyclic peptides, perthamides C and D, were isolated from the tropical marine sponge *Theonella swinhoei*. Perthamide C reduced carrageenan-induced paw edema up to 60% at 300 μg/kg. New perthamides analogues of perthamide C were isolated from *Theonella swinhoei*. Perthamide J (ADAA, replacing Nδ-c-β-OSO3Asn residue in perthamide C) reduced carrageenan-induced paw edema most significantly [122].

The cyclic heptapeptide, stylissatin A (cyclo-(Tyr1-Phe7-Pro6-Ile5-Pro4-Phe3-Ile2)), isolated from the tropical marine sponge *Stylissa massa*, inhibited nitric oxide production in LPS-stimulated RAW264.7 macrophages, with an IC_50_ of 87 μM [123].

Cyclic diketopiperazines, named DKP 1 (L-Hyp-L-Ala) and DKP 5 (L-Pro-L-Ala), isolated from the tropical marine sponge *Callyspongia* sp. showed to stimulate IL-10 production and inhibit TNF-α when murine macrophage-like cell line J774A.1 were treated at 5 μg/mL [68].

In Felician et al. [124], two collagen peptides, CP1 and CP2, derived from the jellyfish *Rhopilema esculentum*, enhanced human umbilical vein endothelial cells (HUVEC) migration at 6.25 μg/mL by increasing the expression of TGF-β1 and b-FGF, factors that serve as signals to recruit inflammatory cells. The in vivo study of Felician et al. [124] on mouse wounds when treated at 0.9 g/kg, showed both collagen peptides to accelerate healing by promoting fibroblast production and chemotactic factors.

In the study of Narayanasamy et al. [125], anti-inflammatory peptide hydrolyzed by trypsin, LGLGAAVL (713.456 Da), isolated from the leg of the marine crab *Charybdis natator* from the Royapuram coastal area in India, showed anti-inflammatory activity by inhibiting albumin denaturation by 91% and preventing destabilization of human red blood cell (HRBC) membranes by 50%. The trypsin-generated peptide exhibited anti-inflammatory activity on LPS-stimulated RAW264.7 macrophages at 50–200 μg/mL by reducing the expression of COX-2 (pro-inflammatory cytokine).

In Wei et al. [126], a cathelicidin peptide of 30 amino acids, named Hc-CATH (KFFKRLLKSVRRAVKKFRKKPRLIGLSTLL), initially isolated from the tropical sea snake *Hydrophis cyanocinctus*, has shown potent anti-inflammatory activity towards LPS-induced MPMs (mouse peritoneal macrophages) by inhibiting 81% of NO production. The mechanism behind this bioactivity was explained by the bindings of Hc-CATH with LPS, therefore neutralizing its toxicity leading to an inflammatory cascade.

*Katsuwonus pelamis*, a species of tuna living in tropical to temperate water, has been studied by Wang et al. [127] for its peptide called SEP (LLFTTQ). This peptide was shown to have anti-inflammatory activity on transgenic zebrafish (by copper sulfate inflammation induction) and in ulcerative colitis (nonspecific inflammatory disease of the colon and rectum) mouse induced by dextran sulfate solution (DSS). For the transgenic zebrafish treated at 500 μg/mL, a decrease in the aggregation of neutrophil granulocytes was observed. For the DSS-induced UC in mice, the combination of a clinical drug, SASP, with SEP at 3 g/100 mL exhibited a regulation of pro-inflammatory and anti-inflammatory cytokines.

**Table 7:** Peptides with anti-inflammatory activity from tropical environments. Abbreviations: LPS for lipopolysaccharide; HUVECs for human umbilical vein endothelial cells; TGFBIp for transforming growth factor β-induced protein; TNF-α for tumor necrosis factor; RA for rheumatoid arthritis; DSS for dextran sulfate solution; UC for ulcerative colitis; SASP for salicylazosulfapyridine.

Protein/Peptide Sequence	Anti-Inflammatory Activity	Active Concentration	In Vitro/In Vivo	Mechanism of Action	Reference
Acrepeptin A	LPS-induced microglial BV-2	IC_50_: 12 μM	In vitro	Inhibition of nitric oxide production	[114]
Acrepeptin C		IC_50_: 10.6 μM			
Aspochracin-type cyclic tripeptide sclerotiotide L	LPS-induced THP-1	10 μM	In vitro	Reduction in LPS-induced expression of IL-10	[113]
Bouillomides A and B	Elastase	IC_50_: 1.9 μM	In vitro	Inhibition of serine protease elastase	[119]
Collagen peptides (CP1 and CP2)	Human umbilical vein endothelial HUVECs	6.25 μg/mL	In vitro	CP1 and CP2 enhanced HUVEC migration by 75.49% and 73.65%	[124]
	Mouse wounds	0.9 g/kg	In vivo (mouse)	Increased signs of re-epithelialization, regeneration and collagen deposition	
Didemnin A	Macrophages RAW264.7 cells	IC_50_: 0.2 µM	In vitro	Oxidative stress reduction	[120]
	Chondrosarcoma SW1353 cells	IC_50_: 0.002 µM		Reduction in inflammatory enzymes expression	
Didemnin B	Macrophages RAW264.7 cells	IC_50_: 0.2 µM		Oxidative stress reduction	
	Chondrosarcoma SW1353 cells	IC_50_: 0.03 µM		Reduction in inflammatory enzymes expression	
Cyclo(L-Pro-D-Val) Cyclo(L-Pro-L-Tyr) Cyclo(L-Pro-D-Leu)	human umbilical vein endothelial HUVECs	10 μM stimulated with 100 ng/mL of LPS	In vitro	Inhibited LPS-induced TGFBIp release and mRNA expression	[112]
	Mouse serum	10 μM	In vivo (mouse)	Reduced TGFBIp release, vascular permeability and leukocyte migration	
Compound 3 (Diketopiperazine dimer)	LPS-induced THP-1	10 μM	In vitro	Reduction in LPS-induced expression of Interleukin-10	[113]
DKP 1	Murine macrophage-like cell line J774A.1	5 μg/mL	In vitro	Stimulation of Interleukin-10 and inhibition of TNF-*α*	[68]
DKP 5					
Grassystatin A	-	IC_50_: 26.5 nM	-	Inhibition of cathepsin D	[117]
	-	IC_50_: 886 pM	-	Inhibition of cathepsin E	
Grassystatin B	-	IC_50_: 7.27 nM	-	Inhibition of cathepsin D	
	-	IC_50_: 354 pM	-	Inhibition of cathepsin E	
HAC	Carrageenan solution	10 mg/kg	In vivo (mouse and rats)	Reduced carrageenan-induced paw edema and inhibited neutrophil migration	[104]
Hc-CATH	LPS-induced MPMs	4 μg/mL	In vitro	Neutralized LPS by binding, blocking its interaction with the Toll-like receptor4/MD2 complex and inhibition of genes that encode inflammatory mediators	[126]
Kempopeptin A	-	IC_50_: 0.32 μM	In vitro	Elastase inhibition	[109]
Largazole	RA synovial fibroblasts	5 μM	In vitro	Inhibition of tissue destruction and inflammatory pathway	[116]
L-G-L-G-A-A-V-L	LPS-induced RAW264.7 macrophages	50–200 μg/mL	In vitro	Reduction in expression of cyclooxygenase-2-2	[125]
Lyngbyastatins 5–7	Porcine pancreatic elastase	IC_50_: 3–10 nM	In vitro	Elastase inhibition	[105]
Lyngbyastatins 8–10		IC_50_: 123 nM, 210 nM, 120 nM		Inhibition of porcine pancreatic elastase	[108]
Molassamide	Elastase	IC_50_: 0.032 μM	In vitro	Protease inhibition	[118]
Perthamide C	Carrageenan solution injection	300 μg/kg	In vivo (mouse)	Reduction in carrageenan-induced paw edema	[121]
Perthamide J					[122]
SEP	Copper sulfate-Induced transgenic zebrafish	500 μg/mL	In vivo	Reduction in the aggregation of neutrophil granulocytes	[127]
	DSS-induced UC in mice	SASP + SEP at 3 g/100 mL	In vivo	Reduction in pro-inflammatory cytokines and increase in anti-inflammatory cytokines	
Somamide B	Porcine pancreatic elastase	IC_50_: 3–10 nM	In vitro	Elastase inhibition	[105]
Stylissatin A	LPS-induced RAW264.7 macrophages	87 μM	In vitro	Inhibition of nitric oxide production	[123]
Thalassospiramide B	Interleukin-5 production inhibition assay	IC_50_: 5 μM	In vitro	Interleukin-5 production inhibition	[111]

### 4.3. Anticancer

Tropical marine ecosystems also harbor organisms producing numerous peptides with antiproliferative/anticancer activities as summarized in Table 8. In a recent study by Najm et al. [128], the anticancer activity of antimicrobial peptides (AMPs) obtained from the mucous of Climbing perch *Anabas testudineus*, a fish which is common in Asia, was investigated. The mucous was extracted, fractionated, and screened for antibacterial activity to confirm the presence of AMPs. The fraction that was cytotoxic to MCF7 (breast adenocarcinoma) and MDA-MB-231 (epithelial human breast cancer cell) was analyzed to identify bioactive peptides. Two of them (AtMP1 and AtMP2) were then produced synthetically. AtMP2 exhibited lower IC_50_ values against both cancer cell lines than AtMP1 and induced cell cycle arrest and apoptosis through regulation of p53, resulting in the up-regulation of the pro-apoptotic gene (i.e., BAX) and the down-regulation of the anti-apoptotic gene (i.e., BCL-2) and ultimately stimulating caspase-3 activation [128].

The cyclic peptide callyaerin G was discovered in 2008 through bioassay-guided fractionation of the marine sponge *Callyspongia aerizusa*, sampled from waters near the Indonesian city of Ambon. This compound was successfully isolated from the ethyl acetate-soluble extract and subsequently evaluated for its biological activity. Cytotoxicity screening revealed that callyaerin G exhibited significant antiproliferative effects against the murine lymphoma L5178Y cell line, demonstrating an ED_50_ of 0.53 μg/mL [129]. Subsequent studies on *C. aerizusa* in 2010 resulted in the discovery of seven additional novel (cyclic) peptides, designated as callyaerins A–F and H. Cytotoxicity evaluations of these compounds against multiple cancer cell lines revealed that only callyaerins E and H had notable antiproliferative activity. These two compounds exhibited potent cytotoxic effects towards the L5178Y murine lymphoma cell line, with ED_50_ values of 0.39 and 0.48 μM, respectively. Structure–activity relationship analysis proposed the cytotoxic potency of the callyaerin series is associated with the number of proline residues present within their cyclic framework [129].

**Table 8:** Anticancer proteins/peptides isolated from marine organisms in tropical environments. Abbreviations: Half maximal inhibitory concentration (IC_50_), effective dose 50 (ED_50_), Lethal Concentration 50 (LC_50_), 50% Growth Inhibition (GI_50_).

Peptide Sequence	Anticancer Activity	Active Concentration	In Vitro/In Vivo	Mechanism of Action	Reference
AtMP2 (TGIATSGLAT FTLHTGSLAPAT)	Breast cancer (MCF7 and MDA-MB-231)	IC_50_: 5.89 ± 0.14 μg/mL for MCF7; IC_50_: 6.97 ± 0.24 μg/mL FOR MDA-MB-231	In vitro	Cell cycle arrest and apoptosis/caspase activation	[128]
Callyaerin G	Murine lymphoma L5178Y cell line	ED_50_: 0.53 μg/mL	In vitro	-	[129]
Callyaerins E and H	Murine lymphoma L5178Y cell line	ED_50_: 0.39; ED_50_: 0.48 μM	In vitro	-	[130]
Sansalvamide A	Pancreatic cancer cell AsPC-1 and CD18 cell lines	Lowest effective concentration of 20–50 μM	In vitro	G0/G1 cell cycle arrest in cells, reduced cyclin D1, cdk4 and 6, and elevated p21 protein expression, and up-regulated cyclin-dependent kinase inhibitors	[131]
Arenamides A and B	Colon carcinoma HCT-116 cells	IC_50_: 13.2; IC_50_: 19.2 μg/mL	In vitro	-	[132]
Lucentamycins A and B	Colon carcinoma HCT-116 cells	IC_50_: 0.20; IC_50_: 11 μM	In vitro	-	[133]
Mechercharmycin A	Cytotoxicity against lung adenocarcinoma A549 and Jurkat leukemia cells	IC_50_: 0.04 μM	In vitro	-	[134]
Thiocoraline	Murine leukemia P388, lung adenocarcinoma A549, and melanoma MEL288 cells	IC_50_: 0.002 μM	In vitro	Induced G1 phase arrest and slowed S phase progression through DNA bisintercalation and DNA polymerase α inhibition	[135,136]
Salinosporamide A	Central nervous system (CNS) SF-539, non-small cell lung NCI-H226, melanoma SK-MEL-28 and breast cancer cells MDA-MB-435	LC_50_ less than 10 nM	In vitro	-	[137]
Similanamide	Breast adenocarcinoma MCF-7, non-small-cell lung cancer NCI-H460, and melanoma cell lines	GI_50_: 115–125 μg/mL	In vitro	-	[138]
Bistratamides H and J	Colon carcinoma HCT-116 cells	IC_50_: from 1.7 and 1 μg/mL.	In vitro	-	[139]
Microcionamides A and B	Substantial cytotoxicity effects towards human breast cancer cell lines (MCF-7 and SKBR-3)	-	In vitro	-	[140]
Bouillonamide	Neuron 2a mouse neuroblastoma cells	IC_50_: 6.0 μM	In vitro	-	[141]
KT2 and RT2	Adenocarcinoma HeLa cells and epidermoid carcinoma CaSki cells	IC_50_: 28.7–53.4 μM; IC_50_: 17.3–30.8 μM	In vitro	Induced apoptotic cell death	[142]
Dolastatin 11	Lung carcinoma NCI-H460 cells, adenocarcinoma OVCAR-3 cells, glioblastoma SF-295 cells, and colon adenocarcinoma KM20L2 cells	nM range	In vitro	Microfilament disruption	[143]
Dolastatins 10	Adenocarcinoma OVCAR-3 cells, glioblastoma SF-295 cell, kidney carcinoma A498 cells, Lung carcinoma NCI-H460 cells, colon ad KM20L2 cells, and melanoma SK-MEL-5 cells	GI_50_: 9.5 × 10^−7^ µg/mL; GI_50_: 7.6 × 10^−6^ µg/mL; GI_50_: 2.6 × 10^−5^ µg/mL; GI_50_: 3.4 × 10^−6^ µg/mL; GI_50_: 4.7 × 10^−6^ µg/mL; GI_50_: 7.4 × 10^−6^ µg/mL	In vitro	-	[144]
Largazole	Mammary epithelial MDA-MB-231 cells	GI_50_: 7.7 nM	In vitro	-	[145]
Kalkitoxin	Breast ductal carcinoma T47D cells	low nanomolar range	In vitro	Inhibition of mitochondrial oxygen consumption at electron transport chain (ETC) complex 1 (NADH-ubiquinone oxidoreductase), preventing the secretion of hypoxia-induced VEGF protein	[146]
Apratoxin A	Colorectal adenocarcinoma LoVo cells and papilloma KB cancer cells	IC_50_:0.36 nM; IC_50_: 0.52 nM	In vitro	Cell cycle arrest, apoptosis, inhibition of phosphorylation of transcription factor STAT3	[147]
Linear polypeptide (PG155)	Human umbilical vein endothelial HUVECs	20 µg/mL	In vitro	Inhibition of VEGF mediated migration and tubulogenesis of HUVECs	[148]
Pardaxin	Human fibrosarcoma HT-1080 cells	IC_50_: 14.52 ± 0.18 μg/mL	In vitro	Apoptosis, by virtue of increase in externalization of plasma membrane phosphatidylserine and chromatin condensation, elevated caspase-3/7 activities, disrupted mitochondrial membrane and pile-up of ROS	[147]
Kahalalide F	Prostate carcinoma DU145 cells, cervical adenocarcinoma HeLa cells, colorectal adenocarcinoma (HT29) cells, and head and neck HN30 cancer cells	-	In vitro	Suppressed the advancement cell cycle to G1 from G0 phase	[149]
Kahalalide F	Hormone-independent prostate tumors, along with neu+ (HER2-overexpressing) breast cancer tumors and neuroblastoma	Maximum tolerated dose in animal models was 300 μg/kg (corresponding to 1800 μg/m^2^)	In vivo	-	[150,151]
Jaspamide or Jasplakinolide	Human prostate carcinoma DU-145 cells, adenocarcinoma PC-3 cells, and Lewis lung carcinoma LNCaP cells	Destroying 1 log of cells with 0.8, 0.3 and 0.07 µM	In vitro	Growth inhibition	[152]
Geodiamolides A, B, H, and I	Breast cancer T47D, MCF-7 and Hs578T cell lines	-	In vitro	Alterations in actin cytoskeleton, Geodiamolide H triggers significant phenotypic changes, reduced cellular migration and invasion in Hs578T cells	[153,154]
Cytotoxic peptide (SBP)	Human carcinoma BEL-7402, human colon carcinoma RKO, human lung carcinoma A549, human glioma U251 and human breast cancer MCF-7 cells.	IC_50_: 7.15 µM; IC_50_: 10.45 µM; IC_50_: 8.41 µM; IC_50_: 6.49 µM; IC_50_: 3.38 µM	In vitro	-	[155]
Microcolins A, B, C, D, E, F, G, H, I, J, K, M, 3,4-dihydromicrocolins A and B, 3,4-dihydromicrocolin D	Human lung cancer H-460 cells	IC_50_: from 6 nM to 5.0 μM.	In vitro	-	[156]
Lyngbyabellin G, O, and P	Human breast carcinoma MCF-7 cells	IC_50_: 120, >160, IC_50_: 9 μM, respectively	In vitro	-	[157]
Lyngbyabellin H and 27-deoxylyngbyabellin	human breast carcinoma MCF-7 cells	IC_50_: 0.07; IC_50_: 0.31 μM	In vitro	-	[157]

Sarcophyton is one of the broadly distributed genera of soft corals in the tropical and sub-tropical oceans, with about 30 species collected and screened for the prospecting of secondary metabolites and particularly fatty acids with cytotoxic activity against brine shrimps. Soft corals are rich in cembranoids (a type of diterpenes) which are present at concentrations up to 5% of dry weight. In vitro cytotoxicity testing has revealed that furano-cembranoids and decariyol isolated from *Nephthea* sp. and *Sarcophyton cherbonnieri* exhibited significant cytotoxicity against several cancer cell lines, including gastric epithelial, liver and breast cell lines [158]. A peptide known as Sansalvamide A isolated from marine fungus of genus *Fusarium*, and its analogues reduced pancreatic cancer cell growth in AsPC-1 and CD18 cell lines. Treatment with concentrations of 20–50 μM for 24 h significantly inhibited DNA synthesis (*p* < 0.001). Using the lowest effective concentration of 20 μM, cell counts at 24, 48, and 72 h showed significant growth inhibition in both cell lines (*p* < 0.001 at 72 h). The treatment resulted in G0/G1 cell cycle arrest, suppressed cyclin D1, cdk4 and 6, and enhanced protein expression of p21 in both cell lines while the cyclin-dependent kinase inhibitors were significantly up-regulated in both CD18 and AsPC-1 cells [131].

Marine-derived actinomycetes (e.g., *Salinispora tropica* and *S. arenicola*) are broadly disseminated in tropical and subtropical ocean sediments [159]. Arenamides A–C, three novel cyclohexadepsipeptides, were isolated from the fermentation broth of the marine bacterium *S. arenicola* obtained from a marine sediment sample collected off the Great Astrolab Reef, in the Kandavu Island chain, Fiji. Arenamides A and B showed modest cytotoxic activity against HCT-116 cells, with IC_50_ values of 13.2 and 19.2 μg/mL, respectively [132]. Marine actinomycetes from tropical waters have yielded promising anticancer peptides. Lucentamycins A-D, 3-methyl-4-ethylideneproline-containing peptides synthesized by *Nocardiopsis lucentensis* from Bahamian sediments, demonstrated cytotoxic activity against HCT-116 cells, with lucentamycins A and B showing IC_50_ values of 0.20 and 11 μM, respectively. The inactivity of lucentamycins C and D suggested that an aromatic ring is required for bioactivity [133].

Mechercharmycins, isolated from *Thermoactinomyces* sp. in sea mud from the Republic of Palau, exhibited structure-dependent anticancer activity. Mechercharmycin A, with its cyclic structure, showed potent cytotoxicity against lung adenocarcinoma A549 and Jurkat leukemia cells (IC_50_: 0.04 μM), while the non-cyclic mechercharmycin B was inactive even at 1 μM, indicating that cyclic structure is crucial for antitumor activity [134].

Thiocoraline, a bicyclic octathiodepsipeptide produced by *Micromonospora* sp., isolated from soft coral off the Mozambican coast in the Indian Ocean [135,160] exhibited strong cytotoxicity against murine leukemia P388, lung adenocarcinoma A549, and melanoma MEL288 cells (IC_50_ = 0.002 μM), with 5-fold higher activity compared to colon adenocarcinoma HT-29 cells (IC_50_ = 0.01 μM) [135]. In colon cancer cell lines LOVO and SW620, thiocoraline induced G1 phase arrest and slowed the S phase progression through DNA bisintercalation and DNA polymerase α inhibition [136].

Salinosporamide A is the most known compound of mixed polyketide-nonribosomal peptides (NRPSs), which serves as potential inhibitor of 20 S proteasome for treatment of cancer [161]. This compound is produced by *S. tropica* strain CNB-392, obtained from shallow sediment (approximately 1 m depth) in a mangrove ecosystem at Chub Cay, Bahamas, as well as by additional *S. tropica* strains CNB-440 and CNB-476, also obtained from Bahamian locations [135,162]. Salinosporamide A exhibited significant in vitro cytotoxicity against HCT-116 cells with an IC_50_ of 11 ng/mL and appears to be highly selective towards NCI- 60 cell line panel. The highest activity was reported against non-small cell lung cancer NCI-H226, central nervous system cancer SF-539, melanoma SK-MEL-28 and breast cancer MDA-MB-435 cell lines (all with LC_50_ values lower than 10 nM) [137].

Similanamide, a novel cyclohexapeptide, was isolated from *Aspergillus similanensis* KUFA0013, a fungal strain associated with the marine sponge *Rhabdermia* sp. collected from coral reefs at the Similan Islands, Thailand, at 10 m depth [163]. Similanamide displayed modest cytotoxicity towards MCF-7, NCI-H460, and melanoma cell lines, with GI_50_ values of 115–125 μg/mL [138]. In addition, marine organisms collected from mesophotic (Twilight Zone, 50–150 m depth) and shallow waters around Guam were evaluated for anticancer potential.

When culturing symbiotic bacteria is challenging or unfeasible, an alternative approach can involve sequencing the biosynthetic genes responsible for producing bioactive peptides and proteins. This strategy is exemplified by the Prochloron symbiont associated with the ascidian *Lissoclinum bistratum* from Bolinao, Pangasinan, Philippines. Bistratamides C and D, isolated from this ascidian, demonstrated moderate cytotoxicity against HCT-116 colon cancer cells [164]. In addition, The Philippine ascidian *Lissoclinum bistratum* yielded six new cyclic hexapeptides, bistratamides E-J. These compounds displayed weak to modest cytotoxicity against the HCT-116 cell line, with IC_50_ values ranging from 1.0 to 28 μg/mL, bistratamides H and J being the most active against HCT-116 cells. Structure–activity relationship analysis revealed that bistratamides containing two thiazole rings exhibited greater cytotoxic activity compared to those with one thiazole and one oxazole ring [139].

Microcionamides A and B, novel linear peptides cyclized through a cystine moiety with a C-terminus blocked by 2-phenylethylenamine, were isolated from the Philippine marine sponge *Clathria (Thalysias) abietina* and their complete structures, including absolute stereochemistry, were elucidated using spectroscopic and chemical analyses. Notably, microcionamide A underwent slow isomerization around the C-36/C-37 double bond when stored in DMSO. Both compounds demonstrated significant cytotoxic behavior against human breast cancer (MCF-7 and SKBR-3) cell lines and exhibited inhibitory activity on *Mycobacterium tuberculosis* H37Ra [140], proposing potential dual therapeutic applications. Microcolins E–M, nine novels linear lipopeptides, along with four known analogs (microcolins A–D), were isolated from the marine cyanobacterium *Moorea producens*. These compounds demonstrated significant cytotoxic activity against lung cancer H-460 cells, with IC_50_ values ranging from 6 nM to 5.0 μM. Structure–activity relationship analysis revealed that structural variations significantly influenced biological activity within the microcolin class of lipopeptides [156].

Bouillonamide, a novel cyclic depsipeptide, along with ulongamide A and apratoxin A, were isolated from the tropical marine cyanobacterium *Moorea bouillonii* collected from New Britain, Papua New Guinea. Bouillonamide exhibited cytotoxicity against neuron 2a mouse neuroblastoma cells with an IC_50_ of 6.0 μM [141]. Ten depsipeptide derivatives (lyngbyabellins) were isolated from *M. bouillonii* from Malaysia and *Okeania* sp. from the Red Sea. These compounds exhibited cytotoxic, antimalarial, and antifouling activities. Against MCF-7 breast cancer cells, lyngbyabellin G, O, and P showed IC_50_ values of 120, >160, and 9 μM, respectively, while lyngbyabellins H and 27-deoxylyngbyabellin A demonstrated potent cytotoxicity with IC_50_ values of 0.07 and 0.31 μM, respectively. Structural analysis revealed that cyclic lyngbyabellins with side chains exhibited the highest cytotoxic activity, while acyclic forms or absence of side chains significantly reduced potency. Additionally, lyngbyabellins A and G delivered powerful antiplasmodial activity against *Plasmodium falciparum* [157].

More than 200 nitrogen containing metabolites have been isolated from two marine cyanobacterial genera Lyngbya and Symploca [165]. Marine cyanobacteria are plentiful in anticancer compounds, exhibiting bioactivity at concentrations as low as the nanomolar (nM) range [166]. These molecules can behave differently based on their therapeutic targets which may be microtubule and actin disrupters, histone deacetylase (HDAC) inhibitors, serine protease inhibitors and molecules that stimulate apoptosis and cell death as discussed in the review by Tan et al. [167].

Microtubule disrupters dolastatins 10/15, initially isolated from Indian sea hare, *Dolabella auricularia*, were then obtained from filamentous marine cyanobacteria which serve as natural diet of the sea hares [168]. Among dolastatins, dolastatin 10, a linear pentapeptide, was the most effective molecule at cancer cell growth inhibition for various human tumor cell lines, with GI_50_ values (in µg/mL) of 9.5 × 10^−7^, 7.6 × 10^−6^, 2.6 × 10^−5^, 3.4 × 10^−6^, 4.7 × 10^−6^, and 7.4 × 10^−6^ for OVCAR-3, SF-295, A498, NCI-H460, KM20L2, and SK-MEL-5, respectively [144]. Multiple analogues of dolastatin 10 have been synthesized. For example, TZT-1027 exhibited angiogenic effects both in vivo and in vitro [169,170]. Dolastatin 11, a hybrid molecule of a polyketide and a polypeptide, is substantially cytotoxic with GI_50_ values in nanomolar ranges against cell lines such as NCI-H460, OVCAR-3, SF-295, and KM20L2 [143]. Dolastatin 11 showed 10–30 times more cytotoxicity than dolastatin 12 in numerous cell lines. Its cytotoxic effects involve microfilament disruption via induction of hyperpolymerization of actin in PtK1 cells which inhibit cytokinesis [171]. In addition, pentapeptide dolastatin-15 which primarily functions as microtubule destabilizer [172,173], also demonstrated HIF-driven antiangiogenic activity and its inhibitory role on HIF- 1α is documented, not only in vitro but also in vivo. Further investigation showed that dolastatin-15 suppressed abnormal transcriptional regulation of HIF-1α genes in the zebrafish model, markedly reducing aberrant vascularization [174].

Marine cyanobacterium *Symploca* sp., from Florida yields another anticancer depsipeptide known as largazole, an HDAC inhibitor. This peptide has shown potential inhibition of the growth of transformed human mammary epithelial cells (MDA-MB-231) at GI_50_ of 7.7 nM and possessed remarkable antiproliferative effects on transformed fibroblastic osteosarcoma U2OS cells (GI_50_ of 55 nM) compared to other natural products including paclitaxel, actinomycin D and doxorubicin [145].

The lipopeptide Kalkitoxin, originally isolated from the tropical marine cyanobacterium *Lyngbya majuscul*, is a neurotoxic agent [146,175]. It was shown to suppress hypoxia-induced activation of HIF-1 in breast ductal carcinoma (T47D) cell in a low nanomolar (nM) range, via the inhibition of mitochondrial oxygen consumption at electron transport chain (ETC) complex 1 (NADH- ubiquinone oxidoreductase). Kalkitoxin successfully blocked the hypoxia-stimulated induction of VEGF or glucose transporter-1 (GLUT-1) mRNA expression in a dose-dependent manner, while also exhibiting antiangiogenic activity by preventing the secretion of hypoxia-induced VEGF protein [146].

Apratoxin A, a cyclodepsipeptide derived from *Lyngbya majuscule*, demonstrated substantial cytotoxic activity, with IC_50_ values of 0.36 nM and 0.52 nM against colon cancer (LoVo) and epidermoid carcinoma (KB) cells, respectively [176]. This compound interfered with receptor tyrosine kinase (RTK) signaling, resulting in the complete inhibition of transcription factor STAT3 phosphorylation [147]. Apratoxin A induced pancreatic toxicity probably associated with the ability of normal pancreas to accumulate the compound, leading to pancreatic atrophy [177].

A linear polypeptide (PG155) was purified from the cartilage of blue shark (*Prionace glauca*) which was able to inhibit VEGF induced migration and tubulogenesis of human umbilical vein endothelial cells (HUVECs). Exposure of HUVECs to 20 µg/mL PG155 markedly reduced the density of migrated cells and complete inhibition of cell migration was achieved at 40–80 µg/mL of PG155. In addition, PG155 significantly inhibited tube formation of HUVECs at 20 µg/mL and dose-dependent effect was seen till the concentration was increased up to 160 µg/mL [148].

Pardaxin, a cell-piercing peptide, isolated from marine fish Red Sea Moses sole (*Pardachirus marmoratus*) exhibited cytotoxic potential against cancer cells [178]. Pardaxin caused anticancer effects via apoptosis, which was supported by a surge in the externalization of plasma membrane phosphatidylserine and chromatin condensation. When cancer cells were treated with pardaxin, a rise in caspase-3/7 activity, disrupted mitochondrial membrane, and accumulation of ROS were observed. Pardaxin caused the inhibition of cell proliferation in human fibrosarcoma HT-1080 cells with IC_50_ of 14.52 ± 0.18 μg/mL at 24 h [178].

Kahalalide F, a depsipeptide, was obtained from marine mollusk (herbivorous species) *Elysia rufescens* found in Hawaii. This peptide was also extracted in inadequate amounts from the mollusk diet, the green algae *Bryopsis* spp. The synthesis of Kahalalide F can be accredited to the mollusk capacity to obtain and retain it from its algal diet [179]. This compound was documented to suppress the progression of cell cycle from G0 to G1 phase in different tumor cell lines such as cervical (HeLa), colon (HT29), prostate (DU145), and head and neck (HN30) cell lines [149]. Studies on the origin of this compound revealed that it has a bacterial origin, from the bacterial symbiont *Candidatus endobryopsis kahalalidefaciens* of green algae. In the natural environment, this molecule is reported to protect the host from predators and is therefore exploited by the predator (*E. rufescens*) for its own defense [180,181]. Kahalalide F exhibited in vitro cytotoxicity against various human tumors, including breast, colon, non-small cell lung, and ovarian tumors, at 1 μM concentration [182]. Cytotoxicity of the compound has been observed in mesenchymal chondrosarcoma and osteosarcoma cells [183]. It has been reported that tumor cells expressing elevated HER- 2/neu and/or HER3 genes exhibit high sensitivity to this compound, which can be associated with caspase-dependent cell death, activity of cathepsin B or D, independent apoptosis and down-regulated AKT signaling [184]. In animal models, hormone-independent prostate tumors, along with neu+ (HER2-overexpressing) breast cancer tumors and neuroblastoma, showed susceptibility to the compound, with an IC_50_ value below 1 μM. Furthermore, the results revealed that the maximum tolerated dose in animal models was 300 μg/kg (corresponding to 1800 μg/m^2^). However, a single 300 μg/kg dose led to organ toxicity during Phase I trials, particularly compromised renal function due to injury in the distal convoluted tubules. Additionally, necrotizing inflammatory activity within the bone marrow and elevated pretrabecular osteocyte proliferation were observed [150].

A tropical marine sponge *Jaspis johnstoni* produces a cyclic depsipeptide known as jaspamide or jasplakinolide with 15 carbon macrocyclic rings containing three amino acid residues [185]. This peptide has produced growth inhibition on human prostate carcinoma (DU-145), PC-3, and Lewis lung carcinoma (LNCaP) cells, reducing 1 log of cells with 0.8, 0.3 and 0.07 µM of the compound in 24 h, respectively [152]. In general, jaspamides have been reported to serve as specialized anticancer agents, stabilizing actin filaments in vitro, while disrupting them and stimulating monomeric actin polymerization into amorphous forms in vivo [186].

Hemiasterlin is a linear tripeptide obtained from the sponge *Hemiasterella minor* and possesses two special amino acids that induced powerful cytotoxicity in the leukemia (P388) cell line [187]. It triggers mitotic arrest and induces apoptosis via the suppression of the mitotic spindle and results in tubulin depolymerization [188]. HTI-286 (aka SPA-110 or taltobulin) is a synthetic analogue of hemiasterlin exhibiting better cytotoxicity than the natural compound on human cancer cell lines and the mechanism of action of both compounds is similar. Preclinical observations revealed HTI-286 suppressed the growth of human tumor xenografts in mice [189]. It has been subjected to phase 1 clinical trials. However, the results appeared insufficient and there were no significant responses in patients with cases of advanced solid tumors, and side effects including nausea, neutropenia, alopecia and pain were observed, leading to trails being terminated [190].

A Gram-positive, rod-shaped bacterium (S-1 of genus *Brevibacillus*), isolated from a marine sediment sample in South China Sea, produced a new cytotoxic peptide (cyclic) with a molecular weight of 1570 Da which was named SBP. MTT (3-(4,5-dimethylthiazol-2-yl)-2,5-diphenyltetrazolium bromide) assay demonstrated cytotoxicity activity in human carcinoma BEL-7402 cells (IC_50_ = 7.15 µM), human colon carcinoma RKO cells (IC_50_ = 10.45 µM), human lung carcinoma A549 cells (IC_50_ = 8.41 µM), human glioma U251 cells (IC_50_ = 6.49 µM) and human breast cancer MCF-7 cells (IC_50_ = 3.38 µM). *Brevibacillus* sp. S-1-produced peptide induced little cytotoxic effects on human normal fibroblast lung (HFL1) cells, suggesting the inhibitory role of this peptide to present some specificity to tumor cells [155].

The cyclic peptides geodiamolides A, B, H and I extracted from *Geodia corticostylifera,* from Brazilian Coast, exhibited antiproliferative effects against breast cancer (T47D and MCF7) cell lines by causing alterations in the actin cytoskeleton. Conversely, primary human fibroblasts and BRL3A remained unaffected after exposure to these peptides, indicating selectivity of these compounds toward malignant cells [153]. Geodiamolide H was further demonstrated to reverse the malignant phenotype of breast carcinoma Hs578T cells, promoting the formation of polarized spheroid-like structures within a 3D environment. Additionally, this marine depsipeptide suppressed migration and invasion of Hs578T cells, seemingly by disrupting the actin cytoskeleton, while non-tumor breast cells (MCF10A) remained unaffected [154]. Furthermore, tamandarins A and B, cyclic depsipeptides exhibiting substantial structural resemblance to the didemnins and therefore proposed to share a comparable mechanism of action, were extracted from a Brazilian ascidian *Didemnum* sp. obtained from Tamandaré, located on the Pernambuco coast in North-Eastern Brazil. Tamandarin A demonstrated high cytotoxicity and marginally greater activity than didemnin B in colony-forming clonogenic assays using human tumor cell lines, with mean IC_50_ values in the nM range [191]. In addition, researchers noted this compound as a potent inhibitor of protein biosynthesis, providing further evidence for a didemnin-like mode of action for these molecules.

## 5. Bioinformatic Tools for Peptide Discovery

Bioinformatic tools integrate sequential, structural, and functional data and may help to better understand how the environmental conditions influence molecular properties in deep-sea, polar and tropical systems, predicting bioactive peptides and structures.

In addition to the bioactivity screening and chemical identification and synthesis, in fact, recent trends also focus on in silico approaches to identify and characterize molecules with beneficial properties (for example, antioxidant) especially with the advent of advanced bioinformatics and artificial intelligence [192,193]. These approaches include bioinformatic tools (e.g., docking or interaction-evaluating tools) able to identify compounds by analyzing available sequences of marine organisms in public databases [192].

For instance, PeptideLocator is a machine learning algorithm to predict bioactive peptides within protein sequences. A recent study focused on the identification of bioactive peptides from Tuna (*Thunnus obesus*) skin [194] by using the BIOPEP website [195], analyzing sequence length, molecular weight, isoelectric point, net charge and hydrophobicity. Bioactive peptides have been generally reported with the N terminal of a non-polar (hydrophobic) amino acid, with sequences of 2–6 amino acids and a molecular weight of 132.05–579.31 kDa. The antioxidant activity of the skin tuna fractions was also confirmed by the DPPH assay [194].

Peptide Ranker (PepRank) is another web-based tool (http://bioware.ucd.ie/~compass/biowareweb/) [196] which predicts the probability of a peptide being bioactive. A recent study applied PepRank to the Atlantic Sea cucumber. The predicted bioactive peptides were then analyzed for antioxidant and ACE inhibitor activities, using the BIOPEP database. The combination of BIOPEP and PepRank tools have also been used to predict bioactive peptides of carp collagen chains [197]. In vitro hydrolysis of the extracted carp collagens was carried out and DPPH radical scavenging activity was used to confirm predictions and validate the antioxidant activity.

In silico tools such as those mentioned in this study (e.g., PepRank, BIOPEP, and SwissADME) play an important role in peptide discovery by enabling rapid screening, identification, and characterization of potential bioactive peptides from protein sources, thereby reducing the time required for experimental work and helping to identify bioactive peptides with potential nutraceutical and pharmaceutical applications [198]. These approaches also allow prediction of toxicity, stability, and drug-likeness, supporting early-stage drug discovery. However, their limitations include strong dependence on existing databases and available information, which may be incomplete, as well as the fact that predictions are only computational and must be validated through in vitro and in vivo studies. Additionally, simulated digestion and bioactivity predictions may not fully reflect real biological conditions, meaning the actual activity and bioavailability of peptides can differ in practice [198].

Another interesting study identified, starting from a cDNA sequence (accession number KU665493), a copper–zinc superoxide dismutase (CuZnSOD) from *Hippocampus abdominalis* (HaCuZnSOD) [199]. The authors characterized in silico its putative protein sequence, homology and evolutionary relationships with orthologs of other species by using ClustalW (https://www.ebi.ac.uk/jdispatcher/; accessed on 20 April 2026) [200], the neighbor-joining (NJ) method in MEGA (ver. 5.0), ExPASy PROSITE Database (https://prosite.expasy.org/; accessed on 20 April 2026) [201], Motif Scan (https://bio.tools/myhits; accessed on 20 April 2026) [202], and I-TASSER (http://zhanglab.ccmb.med.umich.edu/I-TASSER; accessed on 20 April 2026) [203]. Finally, SWISS-MODEL (http://swissmodel.expasy.org/; accessed on 15 April 2026) was used to predict the tertiary structure. In addition, a recombinant enzyme, named rHaCuZnSOD, was overexpressed in a bacterial system and the antioxidant activity evaluated with the xanthine/xanthine oxidase (xanthine/XOD) assay observing higher activities for metal-supplemented rHaCuZnSOD. Authors also studied the peroxidation function of the recombinant enzyme by the MTT assay on THP-1 human cells after H_2_O_2_ treatment and found that cell viability increased in the presence of the enzyme in a dose-dependent manner while intracellular ROS decreased. Overall, authors showed that in silico prediction could identify protein antioxidant characteristics as demonstrated by in vitro and in vivo experiments [199].

Biosynthetic gene clusters (BGC) constitute a particularly valuable bioresource [204]. However, a large number of BGCs remain silent under standard laboratory conditions, and are referred to as cryptic or silent gene clusters [205]. The most used approaches for predicting and annotating BGCs are bioinformatic tools such as antiSMASH (https://antismash.secondarymetabolites.org; accessed on 20 April 2026) [206], NRPquest [207], Pep2Path (https://pep2path.sourceforge.net/; accessed on 20 April 2026) [208]. For example, through bioinformatic analysis using antiSMASH coupled with mass spectrometry, four new compounds were identified as nobilamides from *Bacillus* sp. BCP32, showing antimicrobial activity against multidrug-resistant bacteria [209]. Similarly, in *Rhodococcus* sp. I2R isolated from deep-sea environments, a bioinformatic genome-mining approach combined with mass spectrometry enabled the identification and structural elucidation of 20 new cyclolipopeptides, designated as rhodoheptins, and 33 new glycolipopeptides, designated as rhodamides [84]. Of these, F5 and rhodoheptin mixture showed moderate antiproliferative activity at 500 µg/mL on human A375 melanoma cells.

Despite the increasing number of bioactive peptides identified through both experimental and in silico approaches, their biological activity can vary considerably. Among the marine-approved drugs, the anticancer depsipeptide plitidepsin, commercialized under Aplidin^®^ by PharmaMar, has shown in vitro cytotoxicity over a wide range of cell lines, from hematologic malignancies to solid tumors, with an IC_50_ value below 1 nM [210]. In fact, plitidepsin exhibited an IC_50_ of 0.2 nM in NCl-H460 lung cancer cells, in contrast to other peptides identified such as Marthiapeptide A, Microsclerodermins N and Microsclerodermins O, which exhibited cytotoxic activity against NCl-H460 in the micromolar range (Table 2) [211]. This disparity highlights that while many marine-derived peptides display promising bioactivities, only a limited number reach a potency comparable to clinically approved compounds. Therefore, integrating bioinformatic tools with experimental validation is essential to prioritize the most promising candidates and improve the efficiency of peptide-based drug discovery.

## 6. Conclusions

In general, this review shows how extreme environments can be an extraordinary source of bioactive peptides with significant potential for biotechnological and pharmaceutical applications. The unique environmental conditions of these habitats promote the evolution of biomolecules with distinctive structural and functional properties, such as increased stability and specificity. All this makes them attractive for industrial and therapeutic fields. In particular, this review highlights how the tropical environments harbor about 78% of peptides with antioxidants, anti-inflammatory and anticancer properties discovered in extreme marine environments so far, along with 15% from polar habitats and 7% from deep environments. Despite the promising features, the discovery of novel molecules from extreme environments is still limited by sampling techniques that require further development to enable efficient and reproducible collection of organisms from deep-sea and ice-covered ecosystems. However, sampling technologies can be implemented to shed light on new molecules hidden in organisms living at very deep or very cold (covered by ice) environments. Furthermore, most studies conducted to date have been focused preliminarily on in vitro characterization, showing that the observed properties may depend on the protein sequence, molecular size, hydrophobicity, acyclic forms or absence of side chains. However, the mechanisms of action, in vivo efficacy, methods to optimize extraction and structural characterization are often not clarified. Future studies must focus on the optimization of their isolation, characterization and clarification of activity mechanisms by also structure–activity relationship analyses and in-depth in vivo experiments to confirm the bioactivity and evaluate side effects. Since the major limitation in this field is due to the lack of standardized methods of collection, extraction and purification and the incomplete understanding of mechanism of action underlying compound production, future research should involve optimization study of sampling and isolation strategies in extreme environments and systematic investigation of the relationships between structure and activity of compounds, in addition to the implementation of in vivo studies to complete and confirm the bioactivity found in vitro.

## Figures and Tables

**Table 1:** Comparison between clinically used doses and reference toxicity thresholds. Abbreviations: NIH ODS = National Institutes of Health Office of Dietary Supplements; FNB/DRI = Food and Nutrition Board/Dietary Reference Intakes; EFSA = European Food Safety Authority; EMA = European Medicines Agency; FDA = Food and Drug Administration; ECHA = European Chemicals Agency; UL = Tolerable Upper Intake Level; AI = Adequate Intake.

Compound	Dose with the Strongest Safety Basis	Risk of Toxicity	References
Vitamin C	Adults: 75–90 mg/day recommended intake; smokers: +35 mg/day. Adult UL: 2000 mg/day.	Above 2000 mg/day: mainly diarrhea, nausea, and abdominal cramps; high-dose IV administration represents a distinct pharmacological exposure and is not comparable to supplementation.	NIH ODS; FNB/DRI [39,40]
Vitamin E	EFSA AI: 13 mg/day (men), 11 mg/day (women). EFSA UL: 300 mg/day α-tocopherol; U.S. FNB UL: 1000 mg/day.	Primary signal: bleeding/altered coagulation. In the EU, 300 mg/day represents a prudent upper boundary; the U.S. formal limit remains higher.	EFSA 2015/2024; NIH ODS [41,42,43,44]
*N *-acetylcysteine (NAC)	Standardized medicinal regimens: oral 140 mg/kg loading dose, then 70 mg/kg every 4 h × 17 doses; IV total 300 mg/kg over 20–21 h.	No clear oral toxic dose from supplementation. Main clinical risk is IV: acute hypersensitivity reactions (~17% loading; ~8% adults; ~10% pediatric) and dosing errors may be fatal; animal oral LD50 (rat) > 2500 mg/kg.	DailyMed (ACETADOTE; CETYLEV); ECHA [45,46,47]
Glutathione	Oral: 250–1000 mg/day for up to 6 months; a single 3 g oral dose does not significantly increase plasma levels.	No defined human toxic threshold. Main regulatory concern relates to endotoxin-contaminated IV compounded products rather than intrinsic toxicity; 7 patients reported reactions after 1400 mg IV.	FDA compounding alert [48]
Resveratrol	Most robust regulatory reference: 150 mg/day (EFSA/EU novel food context).	In humans, 2.5–5 g/day for 29 days caused mild-to-moderate gastrointestinal symptoms; no single defined human toxic dose.	EFSA 2016; EU Decision 2016/1190 [49,50]
Quercetin	Common oral doses: 500–1000 mg/day; in 12-week studies, no adverse symptoms or clinically relevant laboratory changes reported.	Clear toxicity signal from IV oncology: dose-limiting nephrotoxicity at 1700 mg/m^2^; renal toxicity observed in 2/10 patients at 1400 mg/m^2^. No formal oral UL identified.	[51]
Melatonin	EMA-approved: Circadin 2 mg (prolonged-release, adults ≥55 years); Slenyto 2–10 mg in pediatric use.	No defined human toxic threshold. The literature reports doses up to 300 mg/day without clinically significant adverse effects; overdose mainly causes somnolence.	EMA (Circadin; Slenyto) [52,53]
Tert-butylhydroquinone (TBHQ)	1–50 µM in vitro and 16.7–25 mg/kg in vivo TBHQ has significant protective effects including the activation of the nuclear factor erythroid 2-related factor 2 (Nrf2) antioxidant pathway, reduction in neuroinflammation following traumatic brain injury and protection of hepatocytes against lipotoxicity	By assessing genotoxicity, TBHQ at 400 mg/kg on mice organs showed DNA damage in stomach cells at 24 h exposure and increased DNA migration in liver and kidney cells due to the formation of ROS [37]. More specifically, acute oral and intraperitoneal lethal doses (LD50) of TBHQ in rats have been documented as 700–1000 and 300–400 mg/kg, respectively [38].	[34,35,36,37,38]

## Data Availability

No new data were created or analyzed in this study. Data sharing is not applicable to this article.
